# Operational research to inform a sub-national surveillance intervention for malaria elimination in Solomon Islands

**DOI:** 10.1186/1475-2875-11-101

**Published:** 2012-03-30

**Authors:** Jo-An Atkinson, Marie-Louise Johnson, Rushika Wijesinghe, Albino Bobogare, L Losi, Matthew O'Sullivan, Yuka Yamaguchi, Geoffrey Kenilorea, Andrew Vallely, Qin Cheng, Andrew Ebringer, Lisa Bain, Karen Gray, Ivor Harris, Maxine Whittaker, Heidi Reid, Archie Clements, Dennis Shanks

**Affiliations:** 1 School of Population Health, University of Queensland, Brisbane, Australia; 2 National Vector Borne Disease Control Program, Ministry of Health, Honiara, Solomon Islands; 3 The Kirby Institute (formally the National Centre in HIV Epidemiology and Clinical Research), University of New South Wales, Sydney, Australia; 4 Australian Army Malaria Institute, Brisbane, Australia; 5 Malaria Drug Resistance and Chemotherapy, Queensland Institute of Medical Research, Brisbane, Australia

## Abstract

**Background:**

Successful reduction of malaria transmission to very low levels has made Isabel Province, Solomon Islands, a target for early elimination by 2014. High malaria transmission in neighbouring provinces and the potential for local asymptomatic infections to cause malaria resurgence highlights the need for sub-national tailoring of surveillance interventions. This study contributes to a situational analysis of malaria in Isabel Province to inform an appropriate surveillance intervention.

**Methods:**

A mixed method study was carried out in Isabel Province in late 2009 and early 2010. The quantitative component was a population-based prevalence survey of 8,554 people from 129 villages, which were selected using a spatially stratified sampling approach to achieve uniform geographical coverage of populated areas. Diagnosis was initially based on Giemsa-stained blood slides followed by molecular analysis using polymerase chain reaction (PCR). Local perceptions and practices related to management of fever and treatment-seeking that would impact a surveillance intervention were also explored using qualitative research methods.

**Results:**

Approximately 33% (8,554/26,221) of the population of Isabel Province participated in the survey. Only one subject was found to be infected with *Plasmodium falciparum *(Pf) (96 parasites/μL) using Giemsa-stained blood films, giving a prevalence of 0.01%. PCR analysis detected a further 13 cases, giving an estimated malaria prevalence of 0.51%. There was a wide geographical distribution of infected subjects. None reported having travelled outside Isabel Province in the previous three months suggesting low-level indigenous malaria transmission. The qualitative findings provide warning signs that the current community vigilance approach to surveillance will not be sufficient to achieve elimination. In addition, fever severity is being used by individuals as an indicator for malaria and a trigger for timely treatment-seeking and case reporting. In light of the finding of a low prevalence of parasitaemia, the current surveillance system may not be able to detect and prevent malaria resurgence.

**Conclusion:**

An adaption to the malERA surveillance framework is proposed and recommendations made for a tailored provincial-level surveillance intervention, which will be essential to achieve elimination, and to maintain this status while the rest of the country catches up.

## Background

Solomon Islands (SI) has had one of the highest levels of documented malaria incidence in the Asia Pacific region, particularly during the period of civil unrest from 1998-2003 [1]. Political stability was re-established in 2003, enabling the Solomon Islands National Malaria Programme to deal with the malaria burden. As a result, the national annual parasite incidence (API) rate (a figure which includes all-cause malaria) decreased from 199 cases per 1,000 population in 2003 to 77/1,000 in 2009 [1,2]. Despite these important gains, the intensity of malaria transmission is highly heterogeneous across eight of the nine island provinces that make up Solomon Islands (SI). For example, passive case detection (PCD) data recorded in 2009 indicate that Guadalcanal and Malaita Provinces had the highest APIs at 155.3/1,000 and 82.9/1,000 population respectively, while nearby Isabel Province had the lowest at 2.6/1,000 population [2].

The success of Isabel Province in reducing malaria transmission, indicated by an API of 64.1/1,000 in 2003 compared to 4.5/1,000 in 2008, identified it as a target for early elimination by 2014 [3,4]. With high malaria transmission in neighbouring provinces and the potential for asymptomatic infections to cause malaria resurgence, a robust provincial level surveillance and rapid response system (hereafter referred to as a surveillance intervention) will be essential to achieving the final step of elimination, and maintaining this status while the rest of the country catches up [5,6]. A qualitative exploration of the feasibility and acceptability of options for surveillance of malaria on Isabel Province was recently carried out to inform the SI National malaria control program surveillance strategy [6]. This study concluded that due to high levels of community engagement in malaria prevention on Isabel Province, and the financial and logistical constraints to introducing formal border screening using rapid diagnostic tests (RDTs), a system of passive case detection (PCD), community vigilance and early treatment-seeking was recommended as the most feasible surveillance option [6]. This approach is heavily dependent on the actions people take when confronted with fever, which may vary from immediate reporting or treatment-seeking to a wait-and-see approach [7]. While this system may be successful in maintaining low levels of malaria transmission in the near term, it is unlikely to be sufficient to achieve the goal of elimination. Over time, with this approach, there could be an increasing risk for malaria resurgence as community enthusiasm wanes as a result of decreasing risk perception [8].

A framework has been developed by the Malaria Eradication Research Agenda (malERA) initiative to assist countries pursuing malaria elimination to determine which are the most appropriate and effective surveillance tools and what indicators should signify a change in strategy as they move along the spectrum of endemicity [5,9]. This framework defines surveillance as 'an intervention' and provides narrative guidance on strategic mixes of surveillance tools and implementation methods such as diagnostics for passive and active case detection, mass screening and treatment, prevalence surveys, case investigation, entomological monitoring, resistance tracking, mapping and stratification, and communication technologies for reporting and rapid response [5]. One tool in the surveillance package not considered in this framework is community participatory surveillance.

Community participatory surveillance has been an important component of a number of communicable disease elimination programmes including the global eradication of smallpox, malaria elimination in Taiwan, the elimination of schistosomiasis in Guangxi Province, China and the programme to eliminate guinea worm in Cameroon [10-14]. Strategies for eliciting and sustaining community participation in a surveillance intervention varied in the different contexts. However, modest cash rewards were a common feature of these elimination programmes and underpinned motivation for early treatment-seeking or participation in surveillance/case containment systems. Cash rewards were often the driving force behind the detection of every last case [10-14].

In the Cameroon guinea worm elimination campaign, as cases of the disease decreased the value of cash rewards increased substantially and each village deemed to be actively participating in prevention activities received an additional cash payment. This incentive strategy and the community engagement in surveillance it stimulated was reported to be a key determinant of the success of the programme [10]. A similar incentive programme was implemented during Taiwan's malaria elimination campaign with a similar effect and contributed to sustained community surveillance and prevention of reintroduction of the disease [14]. Participatory surveillance measures underpinned by modest cash incentives during the elimination and maintenance phase of a programme can contribute to timely identification and containment of pockets of infection, allow targeted intervention rather than blanket coverage and are particularly useful in achieving and maintaining the spotlight on a disease that has ceased to be a priority concern for communities [10-12]. A system of cash incentives to motivate participatory surveillance, while potentially valuable in the elimination and maintenance phases, is not feasible for the control and intensified control phases of a malaria programme in resource-poor settings [15]. Therefore, community participatory surveillance requires varying strategies for implementation according to stages of elimination and should be informed by an understanding of local perceptions and practices in the management of fever and treatment-seeking at health facilities.

The Global Malaria Action Plan calls for intensified control and progressive elimination of malaria from the endemic margins of transmission [16,17]. This has resulted in an impetus in Pacific Island countries to pursue elimination in individual provinces with very low malaria transmission while focussing on intensified control in others. In this context, inter-island travel creates a transient reservoir of infection [17]. Therefore, surveillance as an intervention will need to be tailored to sub-national levels with appropriate strategies designed for different stages along the spectrum of endemicity classifications that exist between provinces [5].

The malERA consultative group on monitoring, evaluation and surveillance recommend that an essential first step in designing a surveillance intervention is a situational analysis [5]. This study was therefore conducted to contribute to a situational analysis of malaria epidemiology and human behavioural factors relating to fever management and treatment-seeking in Isabel Province with the aim of making recommendations for progressing in the medium term to a surveillance intervention capable of achieving and maintaining malaria elimination. In addition, this paper proposes an extension to the malERA surveillance framework [5] by incorporating the valuable role of community participatory surveillance.

## Methods

### Study area and target population

**Figure 1:**
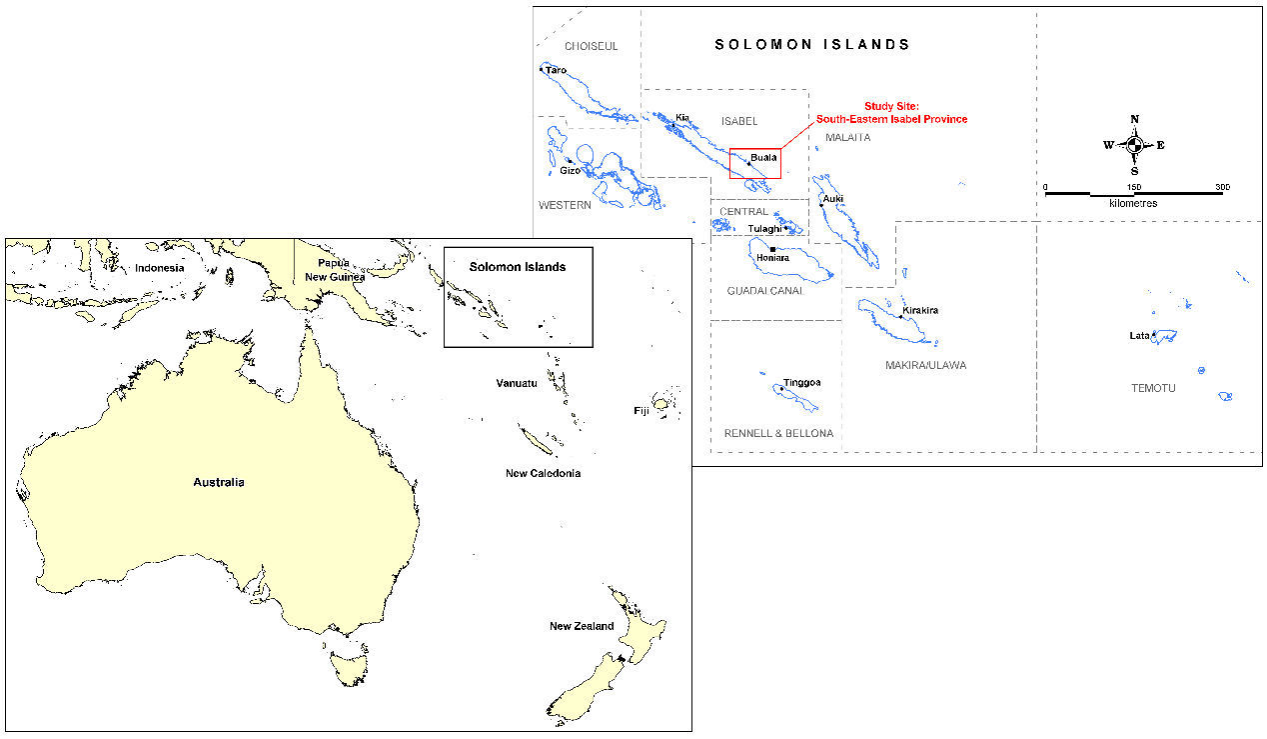
**Map of Isabel Province, Solomon Islands with location of qualitative study site highlighted**.

Isabel Province is located in the north west of Solomon Islands and lies between Choiseul and Malaita Provinces with Guadalcanal Province to the south east (Figure 1). The province is made up of one large island named Santa Isabel (200 km long and 25 km wide), which is the largest and most populous island of the province, and several smaller islands, which are largely uninhabited. The villages on Santa Isabel are predominately located in coastal areas but there are some scattered inland villages particularly in the south east. There are also several logging camps and boarding schools located across Santa Isabel. Although there are a larger number of villages in the south of the main island, the village Kia, with the highest population, lies to the north west. Buala is the provincial capital and a primary first port for travellers by plane and boat.

Successful community engagement and participation has been important to current achievements of the malaria programme in Isabel Province. The collective representatives from the Church of Melanesia (Anglican), Provincial Government and village Chiefs (referred to as the Tripod) along with the Provincial malaria team, and civil society groups such as the Mothers' Union (a worldwide charitable Anglican church group), are noticeably active in working together to achieve community education, awareness and participation in malaria programmes within Isabel Province [6,18]. Methods of control of malaria in Isabel Province have in the past been based on distribution of long-lasting insecticide-treated nets (LLINs) and targeted indoor residual spraying (IRS) for outbreak containment [19]. Surveillance has been based on passive case detection (PCD) [19]. The primary diagnostic tool has been microscopy but since the introduction of the new national malaria treatment guidelines in 2009, RDTs have been used in clinics where microscopists are not regularly available [20,21]. There have also been successful community involvement programmes in vector borne disease prevention such as the "Tidy Village Campaign" which was adopted by almost all villages on Santa Isabel to organize and clean rural villages [6]. Such programmes demonstrate a strong sense of social engagement, which is widely assumed among community members and malaria programme staff to be one of the reasons for successful malaria control in Isabel Province [6].

### Sample size and study population

A mixed method study was carried out on Santa Isabel in late 2009 and early 2010. The quantitative component was a detailed mass blood survey (MBS) of approximately one-third of the estimated total population of 26,221 [4], to determine the prevalence of malaria in Isabel Province. Using an accurate list of villages on Santa Isabel, the spatially stratified survey design used was aimed at achieving uniform geographical coverage of populated areas of the island including coastal and inland zones. This was a modified grid-based design whereby a starting point on the coastline was randomly selected, and subsequent sampling points were evenly placed along the coast, with the nearest village to each sampling point selected for inclusion in the study. In the southern area of the island, where some inland communities were located, a grid was overlain the area in a geographical information system (GIS) and the villages closest to the nodes of the grid were selected for inclusion. Access to the selected villages was achieved by boat and land-based survey teams. Where logistical constraints prevented selected villages being accessed, the next nearest village was selected. A total of 8,554 people from all age groups participated in the MBS. The survey was carried out at the start of the malaria season (October) to coincide with entomological surveys also contributing to the situational analysis of Isabel Province (reported elsewhere) [19].

The qualitative component was carried out during March 2010 to explore local perceptions and practices in the management of fever and treatment-seeking. A particular focus was to understand how individuals differentiated malaria fever from other causes of fever in a low transmission setting and to explore the barriers and motivators for treatment-seeking for fever. Understanding these issues at a local level will be vital to informing the incorporation of a participatory surveillance component in the broader surveillance intervention for malaria elimination. A convenience method was used to select study villages in the south-east region of Santa Isabel Island. Villages were selected in collaboration with key community stakeholders. Village selection aimed to capture a diverse range of community attitudes and perceptions. Selected villages were labelled A, B, C, D and E to maintain participant anonymity in reporting. In each village, focus group discussions (FGDs) of eight to 10 participants were planned as well as key informant interviews (KIIs) with influential community members including village chiefs, teachers, women's group activists, religious leaders, Provincial Council members and health care workers. It was intended that FGDs would be carried out separately with men and women to encourage open dialogue and optimize participation of all members of the focus groups. A total of 130 participants were recruited to the qualitative component of this study.

### Procedure

#### Mass blood survey

Village-level awareness campaigns were carried out prior to the survey with FM radio announcements and promotional visits by the Provincial Malaria Supervisor and a few members of the survey team. The day before a survey team was to visit a selected village, a follow-up awareness visit was made and confirmation of community consent to participate was obtained from village leaders. All intended participants from study villages were instructed to remain in the village on the day of the survey. In some cases, several villages were surveyed at one time by inviting the surrounding villagers to meet in a central village on a single day. With the exception of schools and logging camps, people were surveyed as families to ensure representation of all gender and age groups. On the day of the survey the purpose and procedures of the survey were explained to participants and individual oral consent obtained.

During the survey each participant was interviewed by SI Pijin-speaking local malaria health staff to collect information on their age, gender, primary living location and travel events in the last three months. Data were recorded in logbooks and additionally captured electronically on Personal Data Assistants (PDAs). A quality assurance assessment of the interview information was carried out at the University of Queensland in Brisbane Australia by comparing the two methods of data acquisition. Immediately following the interview, participants had their temperature measured using a tympanic digital thermometer and finger prick blood sample taken to generate thick and thin blood films for microscopic diagnosis. In addition, two blood spots on filter paper were collected for subsequent polymerase chain reaction (PCR) and glucose-6-phosphate dehydrogenase (G6PD) deficiency analyses. Where a participant's temperature was > 37.8°C a rapid diagnostic kit (RDT; CareStartTM malaria HRP2/pLDH Combo Test, Lot#G191R) was additionally used for diagnosis if possible. Rapid diagnostic kits were also used for 914 participants from five villages in the south east of Santa Isabel Island that had been previously identified as areas of local transmission from data provided by the National Vector Borne Disease Control Program (NVBDCP). The G6PD results have been reported elsewhere [22].

Giemsa-stained thick and thin blood films were read on the day of collection by experienced microscopists from the NVBDCP and the Australian Army Malaria Institute (AAMI). These microscopists had been previously certified as WHO-accredited level 1, 2 or 3 malaria microscopists. A subset of slides (all positives and a random 10% of negatives) were checked as a quality assurance procedure by WHO accredited Level 1 or 2 malaria microscopists. Molecular diagnosis using PCR was carried out at AAMI in Brisbane, Australia from filter paper samples. The samples tested using PCR included those from microscopy positive, RDT positive and febrile subjects, 10% random samples and all samples from the six villages where the index malaria cases or infected mosquitoes had been identified. A total of 2,001 samples were tested and each sample was subjected to a multiplex PCR based on the method and procedure outlined elsewhere [23,24].

#### Qualitative study

FGDs and KIIs were triangulated with informal field observations recorded by trained field research officers. The field officers were trained in qualitative research methods, research logistics, ethical considerations, equipment use and data management and analysis, and were supervised by senior social scientist and public health researchers from the School of Population Health, University of Queensland. All FGDs were carried out in SI Pijin and where participants felt more comfortable speaking in local dialect, interpreters were used to ensure all participants could contribute to discussions in a meaningful way. Semi-structured interview guides were used to facilitate discussions which were digitally recorded with consent. Further details of the methodology of the qualitative study have been reported earlier [6].

### Data analysis

#### Mass blood survey

Malaria prevalence determined by microscopy was calculated as the number of microscopy positive samples divided by the number of samples examined by microscopy × 100%. Malaria prevalence determined by PCR in the random sample subset was calculated as the number of PCR positive samples in the random sample set divided by the number of PCR tested random samples × 100%. Estimated malaria prevalence by PCR was calculated as the sum of PCR positive samples (in microscopy positive, RDT positive and microscopy positive groups, six villages and estimated positive numbers in remaining samples derived from the prevalence in the 10% random set) divided by the sum of total samples (microscopy positive, RDT positive, microscopy positive, six villages and remaining samples) × 100%.

#### Qualitative study

Digital recordings of the FGDs and KIIs were transcribed and translated from Pijin to English by local research officers. Data were entered into NVivo 8^® ^software (QSR International Pty Ltd, Australia). Engagement with the data produced a coding key which was agreed by the data analysis team and subsequently utilized by the primary coder. Data was then organized into identifiable themes and patterns and subjected to thematic analysis. Areas of consensus and divergence were identified and a 'realist method' used to understand participants realities, experiences and meanings [25]. Issues arising with regards to coding and thematic analysis were discussed and resolved collaboratively between Australian and SI investigators.

### Ethical aspects

This research was approved by the National Health Research Ethics Committee, Solomon Islands, the School of Population Health Ethics Committee, UQ, Australia (Approval No. GK050110) and the Australian Defence Human Research Ethics Committee (Protocol no. 505/07). Informed consent was obtained from all participants. For the mass blood survey each participant was asked by the SI Pijin speaking health worker on the survey team if they would provide consent to have their blood taken. Verbal informed consent was recorded at the time of the sampling in a log book. For the qualitative study, individual informed consent (written or witnessed thumb print) was obtained from all participants prior the FGDs and KIIs following a verbal and written explanation of study aims and procedures. Consent was obtained separately for participation and for taping of interviews. The privacy of participants for both surveys was preserved by allocating numbers to participants in the mass blood survey and not identifying individuals by their full names in the qualitative transcripts. All data was securely stored during the field activities and subsequently on return to the University of Queensland and the AAMI. Data shared with the Solomon Island Ministry of Health and Medical Services was de-identified.

## Results

### Results of mass blood survey

In total, 8,554 people from 129 villages (including two logging camps and two schools) were surveyed over a four-week period covering all areas of the province. Only one village could not be accessed due to poor weather conditions. This was replaced with another nearby village. Participants covered all age groups (Table 1) and were of mixed gender (54.2% female and 45.8% male). Almost 50% of subjects were children under 14 years of age.

**Table 1:** Age distribution of participants in mass blood survey, Isabel Province, Solomon Islands, 2009

Age	N	%
0-14	4,215	49.3
15-29	1,641	19.2
30-44	1,442	16.9
45-59	735	8.6
65+	519	6.0

Total	8,554	100

#### Malaria prevalence determined by microscopy and PCR

Only one positive subject was found to be infected with *P. falciparum *(96 parasites/μL), by microscopy out of 8,554 people surveyed, giving a prevalence of 0.01% (95%CI:0.008-0.012). The positive subject was a child (aged between 10-14 years) from the north west of Santa Isabel who had not travelled recently. This child was carrying only gametocytes, which indicated that he had the infection for some time and subsequent PCR confirmed the *P. falciparum *infection. None of the other participants sampled from the same village was found to be positive by microscopy or PCR analysis.

**Figure 2:**
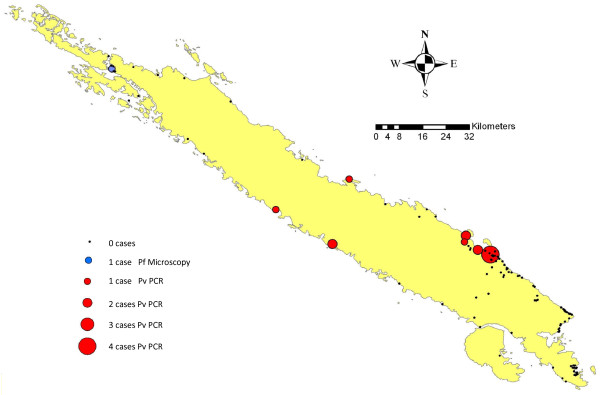
**Map of Santa Isabel Island showing villages surveyed and the location of *Plasmodium falciparum *and *Plasmodium vivax *cases identified by microscopy and PCR**.

PCR analysis of the 2,001 samples from the survey revealed an additional 13 subjects carrying *Plasmodium vivax *parasites (Table 2): one from the febrile group (resident of village 5), two each from villages 1, 2 and 4, three from village 5 and three from the 10% random sample. The PCR determined malaria prevalence in the random sample subset was 0.55% (3/541) (95%CI:0.508-0.593) at the time of blood survey. Assuming that this rate applies to the remainder of the samples that were not PCR tested and combining this with the PCR prevalence of the six villages, the estimated malaria prevalence at the time of survey would be 0.51% (95%CI:0.499-0.521), five-fold higher than that determined by microscopy. The malaria-infected subjects are scattered across the island with a wide geographical distribution (Figure 2).

**Table 2:** Number of PCR positive samples in different locations and groups

Sample source		Total	Febrile	*Plasmodium* *falciparum *+	*Plasmodium* *vivax *+
Villages	1	331	7	0	2
	
	2 (school)	57	6	0	0
	
	2	200	0	0	2
	
	3	125	1	0	0
	
	4	215	2	0	2
	
	5	339	4	0	4*
	
	6	35	2	1	0

10% random sample subset across all villages		541	2	0	3

Febrile from other villages		149	149	0	0

RDT positive		9	0	0	0

Total		2,001	173	1	13

Note: *This number includes one of the 173 febrile subjects who was identified as positive for *P. vivax*.

A total of 972 participants were tested for malaria with RDT kits including 65 febrile subjects and all subjects from five villages (Gnulahage, Sigana Island, Ligara, Tanamuki and Tausese). Nine subjects tested positive for malaria with RDTs in the field. However, in subsequent PCR analysis none of these RDT positive samples tested positive and, therefore, the nine RDT positives were considered false positives (RDTs used were subsequently found to be faulty). One hundred and seventy three participants had temperatures greater than 37.8°C at the time of survey. PCR analysis of the filter paper blood spots taken from these febrile participants found only one tested positive for *P. vivax *from village 5 in the south of Santa Isabel. Although negative at the primary microscopy examination, a re-examination of the blood smear by expert microscopists identified *P. vivax *in this subject with a parasite density of 47P/μL.

#### Travel history

Ten percent of the survey participants indicated that they had travelled out of Isabel Province at some time during April to October of 2009. The most visited province was Guadalcanal, with 93% (767/827) of travel, followed by Western Province at 2% (16/827) and Malaita Province at 1% (7/827). No statistically significant difference was found in reported travel outside Isabel between males and females (53% male and 47% female, p value = 0.0812). People travelled from 59% of the villages surveyed. These villages were evenly distributed geographically around the province. Higher percentages of people travelling outside of Isabel Province were observed in Bokolo (33%), Koviloko (30%), U'uri (29%), Tusa (23%), Kmaga (23%) and Buala (18%). Children from Jejevo School (Buala), Guguha School and Hofi School also travelled outside the province (28, 11, and 25% respectively). Of 44 people surveyed from Matamata logging camp 30% travelled outside the Province. Despite this, the survey showed none of the participants testing positive for malaria by microscopy or PCR analysis had travelled outside Isabel Province in the previous three months.

### Results of qualitative investigation

A total of 13 FGDs and 22 KIIs were conducted over the four-week study period. The number of participants in each FGD ranged from six to 12. Although it was intended that FGDs were conducted separately with men and women, two mixed gender FGDs were carried out (one with health workers and the other with the Isabel Provincial Assembly). The mixed gender format of these two FGDs did not appear to inhibit open dialogue. The villages were generally homogeneous in their views regarding the key issues being investigated. Where participant responses differed between study villages or gender they are highlighted in the results presented, otherwise similar responses have been reported collectively.

#### Perceptions of malaria

Although some participants reported malaria to be a significant public health problem causing illness and death in the past, most participants did not perceive malaria to be a current problem in their own village or in Isabel Province as a whole. There was variation in reporting on how recently malaria was a problem between participants from different villages. However, recollections of mortality from malaria usually dated back more than a decade. Some participants in their mid- to late-20 s claimed never to have had experienced malaria illness.

Risk of malaria was commonly perceived as external. That is, the source of transmission was perceived to be from nearby endemic provinces and that population movement between Isabel and surrounding provinces, particularly Guadalcanal, was thought to increase the risk of malaria transmission. Loggers and local businessmen travelling to and from Isabel Province were singled out as high-risk individuals for carrying 'Guadalcanal' malaria. Students from other provinces coming to study or returning home to Isabel Province were also considered high-risk individuals. These perceptions are in contrast to findings of the mass blood survey, which suggest indigenous malaria transmission in Isabel Province.

#### Differential diagnosis of fever

Most participants could correctly identify an array of malaria symptoms which were reported as high fever with any combination of headache, shivering, feeling cold, body/joint aches and pains, nausea, vomiting, diarrhoea, dizziness, body weakness, bitter taste and a loss of appetite. A few participants mentioned that malaria could be present with non-specific symptoms (like abdominal pain or feeling "delusional"), and a few reported the observation that the symptoms of malaria have changed over time.

The majority of FGD participants indicated they could distinguish between malaria fever and fever from other causes based on past experience of malaria. The few participants who had never experienced malaria reportedly relied on cues based on observations of relatives who had had malaria in the past.

'... you suspect malaria fever when fever is very high, your head feels warm but your feet are cold... and malaria fever makes me "karange" (mental)' (Female participant, FGD, village D)

*'... once the body is very cold inside and hot outside I know it is malaria... this kind of feeling will make you want to sit in the sun or close to the fire. Sometimes covering you with two to three blankets is still not enough...' *(Female participant, FGD, village B)

Despite claims of being able to distinguish malaria fever from other causes of fever, the most commonly reported causes of fever in the communities were the common cold, flu, pneumonia, diarrhoeal illness and 'fever from boils'. Malaria fever was spoken about as a thing of the past. Some participants reported that 'fever thought to be malaria' always turns out to be pneumonia. Health workers also spoke of the past when malaria was distinguishable clinically from other illnesses that caused fever. However, they reported that in recent times RDTs or microscopy are needed to rule out a diagnosis of malaria. Nonetheless, among health workers interviewed, malaria is reportedly still suspected when patients arriving from neighbouring provinces present with fever.

#### Treatment-seeking

There were no differences between men and women or participants from different villages in their reporting of usual treatment-seeking practices for fever. Most participants indicated that early treatment-seeking for fever at a health facility was important, particularly for children. However, some admitted that they and others sought treatment only when home remedies failed to resolve symptoms or their condition deteriorated. This reportedly could take up to three days from the onset of fever. A few participants reported that their first response to fever was to have a cold shower, perform cold sponging, use traditional herbal remedies, participate in prayer with church leaders or self-medicate using paracetamol or aspirin available at home or from neighbours.

*'... when it comes to healing this kind of fever... you will hear people... they won't go straight to the hospital... they will go first to the custom man, after that then they look for the custom medicine and if that doesn't work... they head to the hospital but by then it's all very serious*. (Male participant, FGD, village A)

A few participants reported using these varied biomedical and traditional remedies interchangeably. Additional reasons for delayed treatment-seeking reported by some participants were a lack of transport, lack of finance for transport, bad weather and waiting until daybreak.

*'... for me personally when I come across really big illness like malaria, I have to be satisfied so I go through hospital, church and custom (traditional treatment) before I am confident... I have to take three treatments. Hospital, custom and church that's what I do...' *(Male key informant, village A)

#### Motivation for ongoing community participation in the malaria programme

The participants revealed that strong bonds exist within and between villages and within Isabel Province as a whole. The Tripod structure has a very strong influence on communities and the spirit of togetherness in community tasks and activities was highlighted by the participants and exemplified by the success of the Tidy Village Campaign. This campaign is reported to be under the direction of the chiefs in association with village health committees. Participants reported that there are health committees within each village that work with the provincial malaria team under the approval of the village chief. Participants particularly highlighted the influence of the church on the lives of people in Isabel Province. Organizations within the Church (e.g. the Mothers' Union) assist provincial malaria workers to raise awareness, distribute LLINs and encourage their use. Some participants recognised the importance of the NVBDCP continuing such efforts to collaborate with these stakeholder groups in Isabel Province. Many study participants reinforced the importance of maintaining efforts against malaria and complacency was recognised as risky.

*'Malaria is like a spring; once it goes down if you relax it'll shoot up again' *(Male key informant, village A)

Motivation to take ongoing action was based on the sustained engagement of provincial malaria workers with communities and their efforts to build partnerships to prevent malaria.

'Every Friday when the chief beats the drums or makes noise with the haler and puts up the "malaria flag" the whole village will work with the village health members in cutting grass, digging drains, and so on... sometimes the provincial malaria workers also work with us telling us what to do...' (Male participant, FGD, village B)

## Discussion

These studies have contributed to a situational analysis of malaria transmission in Isabel Province which has been advocated by the malERA group as an important first step in determining an appropriate surveillance intervention and indicators for transitioning to alternative surveillance strategies [5]. Analysis of the qualitative findings highlight that while community cohesiveness and cooperation fostered by the influential Tripod structure will be valuable in engaging communities in surveillance interventions, this alone may be insufficient in the final step of achieving and maintaining malaria elimination. Despite many participants highlighting the importance of maintenance efforts against malaria, early warning signs are emerging that the current community vigilance approach to surveillance will not be effective in the medium to long term. This is because malaria is already being seen as a disease of the past and not currently a problem. In addition, unlike diseases such as smallpox and guinea worm, malaria lacks visibility. Fever severity is being used by individuals as an indicator for malaria. However, in light of the MBS findings of primarily asymptomatic, low parasitaemic cases, this may be an insufficient trigger for timely treatment-seeking and case reporting. In addition, the qualitative study highlighted community perceptions of malaria being imported and the risk as being externalised as a consequence of travel to other Provinces. This is contradicted by findings of the MBS, which suggested indigenous malaria transmission in Isabel Province. As a result of these perceptions of risk and triggers to suspect malaria, treatment-seeking for fever at a health facility is delayed for up to three days and used as a last resort when home remedies and traditional medicines fail to resolve symptoms.

The qualitative findings are consistent with studies carried out elsewhere in the south west Pacific and beyond where sustained low transmission has resulted in waning vigilance and delayed treatment-seeking for fever [8,26,27]. An important lesson arising from previous successful elimination campaigns is the vital role of a participatory surveillance intervention, and in particular, the inclusion of modest incentives to underpin and maintain motivation for early treatment-seeking and case reporting [10-14,28]. However, in resource-poor countries such as SI, the introduction of a widespread or prolonged cash incentive-driven participatory surveillance intervention is not feasible. Therefore, alternative strategies for community engagement in malaria surveillance are required with modified approaches across the endemicity spectrum. In addition, longitudinal community-based qualitative monitoring at sentinel sites for waning motivation for community participation in the surveillance intervention may be an important tool during the final push to achieve elimination [8].

The MBS revealed malaria prevalence to be very low at the time of survey. The PCR estimated prevalence of 0.51% confirms the findings of the 2009 malaria report by the National Vector Borne Disease Control Program and suggests that malaria elimination may be technically feasible on the island. However, detection of malaria-infected individuals in low transmission areas is a major challenge to malaria elimination programmes. This study highlighted the additional difficulties in detecting people who carried a low density of parasites and were mostly asymptomatic using available diagnostic tools of microscopy and RDTs. Similar to that reported for Temotu, Solomon Islands [29], fever was not a good indicator of malaria because only one of the 173 febrile patients was PCR positive for malaria. As expected, and reported elsewhere [29-31], microscopy was less sensitive than PCR at detecting very low parasitaemia, identifying only one of the 14 PCR positive subjects. Re-examination of PCR positive slides by microscopy identified an additional positive subject who had a parasite density of 47 parasites/μL. It is highly likely that parasite densities in all these PCR-positive, microscopy-negative individuals were < 100/μL and hence not detected by the expert microscopists. The performance of RDTs in detecting these low density infections is also not satisfactory although this product has been shown to readily detect parasite density of 200/μL at the WHO product testing [32]. Novel molecular diagnostic tools are therefore needed for surveillance in this setting. However, if used in combination with other diagnostic and surveillance measures, RDTs could remain a useful tool for malaria elimination [33].

The MBS reported here revealed no association between malaria positive cases (by microscopy or PCR) and travel outside Isabel Province. This is additionally supported by a recent MBS of secondary schools and logging camps in Isabel Province in 2011 as part of active surveillance of population groups that travel frequently in and out of the Province, which revealed no microscopy positives in 1,000 slides taken from secondary schools and very few positives (two *P. falciparum *and two *P. vivax *infections) out of the 668 slides taken from logging camps [34]. These findings point towards the possibility of a very low level of indigenous transmission still occurring within Isabel Province, making rigorous active case detection an immediate and absolute necessity in the final push towards eliminating the last cases of malaria. In addition, since even low undetected importation of malaria can trigger important local transmission [35], these activities should be complemented by ongoing efforts to detect and prevent malaria from being introduced into Isabel Province from neighbouring high prevalence provinces. Therefore, in Table 3 an adaption to the malERA framework for surveillance is proposed which incorporates the community participatory surveillance component and sets out varying strategies for effective surveillance interventions across the endemicity spectrum. This adapted framework is intended only to provide a guide for programmes and, as suggested by the malERA group, surveillance interventions should be tailored to local contexts based on a situational analysis.

**Table 3:** Tailoring malaria surveillance to phases along the spectrum of endemicity*^§^:

Surveillance tools	Control phase Intense stable endemic (hyper-holoendemic)	Intensified Control phase Moderate stable endemic (mesoendemic)	Elimination phase Unstable endemic (hypoendemic/ outbreak risk)	Elimination phase Non-endemic, disease free (outbreak risk)
**Case finding** **and treatment**	PCD and standard treatment protocol	PCD and standard treatment protocol PLUS Baseline MBS with radical treatment (with G6PD testing or DOTs) and transmission risk mapping/coverage mapping	PCD and standard treatment protocol PLUS 'Reactive' case detection/case investigation with targeted/localised MDA (particularly in context of low parasitaemia/low prevalence)	PCD and standard treatment protocol PLUS 'Reactive' case detection/case investigation with targeted/localised MDA PLUS Maintenance phase periodic survey of age stratified serology to confirm disease free status

**Entomology**	Resistance monitoring and evaluation of effectiveness of vector control interventions	Resistance monitoring and evaluation of effectiveness of vector control interventions	Regular entomological surveys to monitor resistance development, effectiveness of integrated vector control interventions and vector behaviour	Maintenance entomological surveys

**Border** **surveillance**	Not indicated	Not indicated	Targeted sub- national border surveillance based on transmission data (i.e. use only if transmission is a result of mobility and repeated re-introduction rather than from persistent indigenous foci)	National level border surveillance

**Community engagement**	Education and engagement of influential community-based structures to encourage early treatment-seeking for fever	Education and engagement of influential community-based structures to encourage early treatment-seeking for fever PLUS participatory vector surveillance activities	'Eyes and ears' approach for early treatment-seeking/ case reporting PLUS participatory vector surveillance PLUS Feedback of surveillance data to communities to maintain motivation (consider incentives for community case identification in final push to eliminate**)	Incentives for case identification** PLUS participatory vector surveillance during elimination and maintenance PLUS Feedback of surveillance data to community (+/- SMS technology)

*Endemicity classifications adapted from Hay *et al*. 2008; ^§^Surveillance tools recommended across the spectrum of endemicity adapted from recommendations by malERA Consultative Group on Monitoring, Evaluation, and Surveillance; MBS - Mass blood survey; DOT - Directly observed treatment; MDA - Mass drug administration; PCD - Passive case detection; ACD - Active case detection; RDT - Rapid Diagnostic Test; G6PD - Glucose- 6 -phosphate dehrogenase; SMS - Short Message Service; ** Incentives may be non monetory

### Recommendations for a surveillance intervention for Isabel Province, Solomon Islands in the push to eliminate malaria

Isabel Province has succeeded in controlling but not eliminating malaria. This situational analysis with qualitative and quantitative components has highlighted a number of key issues that need to be addressed by an appropriate surveillance intervention to achieve elimination at the sub-national level. These issues include: delayed treatment-seeking for fever; perceptions of malaria being primarily imported whilst data indicates primarily indigenous sources of transmission; low malaria transmission dispersed across the island; asymptomatic cases; and inadequate locally available diagnostic tools to detect low density parasitaemia. Because malaria is no longer a significant public health problem in Isabel Province in terms of morbidity and mortality, resource-intensive periodic MBSs with PCR testing, as conducted in this study, are not a feasible or affordable option as part of a sustainable surveillance intervention. Such a sustained intervention would need to be implemented over potentially lengthy periods to clear malaria from the other parts of Solomon Islands.

A mathematical model developed by Karl *et al*., adapted the classical Ross-Macdonald model of malaria transmission and accounted for low-level gametocytaemia in the population [36]. Using this adapted model the authors predicted persistent low-level transmission even with high LLIN coverage and usage [36]. In addition to these issues, a previous study has also identified a 20.3% prevalence of G6PD deficiency in the population of Isabel Province, which included a 6.9% prevalence of severe deficiency that would predispose people to primaquine-induced haemolysis (WHO Class I-II) [22].

In light these issues, despite the high level of community engagement in malaria prevention interventions, the current surveillance system of PCD, community-based vigilance and early treatment-seeking for fever is unlikely to be sufficient to support malaria elimination in Isabel Province beyond the short term. A more active surveillance intervention will be required which should harness the benefits of community engagement that have already been achieved in the province. Consistent with the adapted malERA framework (Table 3) this surveillance intervention should consist of the following key elements:

1. **Case finding and treatment/rapid response**: Transmission interruption will require more active strategies to supplement PCD for identification and elimination of malaria foci. 'Reactive' case detection entails rapid response to case reporting that includes case investigation in the area where a parasitaemic individual is found plus targeted mass drug administration and aggressive vector control. In the Isabel context this would mean using any parasite positive case as an indicator of local transmission and concentrating epidemiology and control resources on such remaining foci. Although this approach will not find isolated asymptomatic parasitaemic persons, it is usually not possible to do the multiple rounds of ACD to find such hidden remnants until at least one symptomatic case occurs in the area. This approach is advantageous as it is less resource-intensive than ACD or aggressive ACD but is an active surveillance intervention tool [37,38]. Procedures regarding how many persons to treat within the radius of the presumed transmission area are yet to be determined; as is the type of treatment, the decision to screen for G6PD deficiency and whether to include community-based directly observed treatment. The feasibility, effectiveness and acceptability of the various ways and means of applying this approach needs to be practically worked out through field evaluations. However, it is anticipated that using positive cases as an indicator of where malaria transmission is likely to still be occurring and implementing a rapid aggressive response will gradually eliminate the last parasites from the human hosts in a defined area such as an island.

2. **Entomology**: An entomological survey carried out in 2009 found *Anopheles farauti *to be the primary vector transmitting malaria on Santa Isabel [19]. This vector was found mostly in large brackish swamps in coastal regions where the majority of the population are located [19]. To achieve elimination in Isabel Province, it was therefore recommended larval control be implemented in coastal villages where large, favourable sites allow for high numbers of *An. farauti *breeding [19]. With the presence of additional but inefficient vectors capable of transmitting malaria and the risk of a shift in biting behaviour of *An farauti *to early night outdoor feeding, vector monitoring is also indicated as a priority in Isabel Province [19]. Such larval control and entomological monitoring activities could be feasibly incorporated into existing community-led actions such as the 'Tidy Village Campaign.'

3. **Border surveillance**: A formalized border surveillance system for Isabel Province is not currently indicated because data suggests primarily indigenous sources of transmission. However, a targeted sub-national border surveillance system may need to be considered in the future should data indicate transmission arising from mobility and repeated re-introduction.

4. **Community engagement**: A continued focus on education, promotion of preventative practices, case reporting and early treatment-seeking for fever, incorporation of vector surveillance and larviciding activities into the 'Tidy Village Campaign,' and potential engagement of community members in delivery of targeted MDA with directly observed treatment is recommended as part of the surveillance intervention for Isabel Province. In addition, to address misconceptions of risk, potential waning of enthusiasm for early treatment-seeking for fever and case reporting a mechanism for feedback of surveillance data to the community level should be introduced. Two-way data flow, clearly defined channels of communication (i.e. between the malaria team and aid posts, health clinics, schools, churches), and timely decentralized decision-making and response to cases has previously been reported as an effective mechanism for sustaining active community participation in a surveillance intervention [8,38-40]. It has also been suggested that this mechanism should not focus on a single disease but should form part of an integrated surveillance system that includes other locally important communicable diseases [39].

Despite these recommendations having been informed by a situational analysis of qualitative and quantitative characteristics of the context of Isabel Province, very little evidence exists that would predict the potential reliability and effectiveness of the proposed combination of tools that constitute this participatory surveillance intervention for malaria elimination in a resource poor setting. The implementation and diligent evaluation of the effectiveness and sustainability of this surveillance intervention in Isabel Province will therefore be vital not only for moving forward with elimination but also to prevent resurgent malaria and its devastating effects seen elsewhere [41,42]. This work has highlighted the challenges with surveillance efforts, even on a small island in the south west Pacific, and emphasizes the need for the development of robust tailored surveillance interventions, particularly in light of recent estimates of higher than anticipated levels of global malaria mortality.

### Limitations of the study

Any cross-sectional survey for malaria may or may not reflect the time of greatest malaria transmission. Sensitivity of the tests used have their limitations, especially with low-level parasitaemia. All FGDs where conducted in SI Pijin. Non-Pijin speaking facilitators conducted KIIs in English. Where key informants where not able to express views and opinions to their full extent due to incomplete grasp of English language, they were encouraged to respond in Pijin which was later translated to English by a fluent Pijin speaking facilitator. The potential loss of nuances that can occur through the direct transcription and translation of recordings from Pijin to English was minimized by the inclusion of a native speaker on the qualitative data collection and analysis team [43].

## Conclusion

The success of Isabel Province, Solomon Islands in reducing malaria transmission to very low levels provides optimism that elimination may be achieved eventually. However, the current community vigilance approach to surveillance is unlikely to be sufficient to achieve the goal of elimination. Over time, with this approach, there could be an increasing risk for malaria resurgence as community enthusiasm wanes. High malaria transmission in neighbouring provinces and the potential for local asymptomatic infections to cause malaria resurgence highlights the need for sub-national tailoring of surveillance interventions. The framework developed by the malERA group assists countries pursuing elimination to determine appropriate surveillance tools for various phases along the spectrum of malaria endemicity. As recommended by the malERA group, this study has contributed to a situational analysis of malaria in Isabel Province to inform the design and implementation of an appropriate surveillance intervention that will be able to achieve the final step of elimination, and maintain this status while the rest of the country catches up. In addition, this paper proposes an extension to the malERA framework to incorporate an important but omitted tool in the surveillance arsenal; that of community participatory surveillance.

## Competing interests

The authors declare that they have no competing interests.

## Authors' contributions

AV, MLJ, MO, YY, GK, JA, RW, HR, AE, AC, MW, LL, AB and DS conceived and designed the quantitative and/or qualitative components of the study and/or supervised fieldwork. LB, KG and QC modified and performed methodologies for PCR and quantitative data analysis. Qualitative data analysis was carried out by YY, MO, GK with guidance from JA. Manuscript was written by JA with contributions from MLJ, IH, MW, AV, AC, AE, DS, RW, LB, KG and QC. All authors have read and approved the final manuscript.

## Supplementary Material

Additional file 1 **List of staff whose contribution to the mass blood survey is acknowledged and appreciated**. Click here for file
