# Supplementary material for: Operational research to inform a sub-national surveillance intervention for malaria elimination in Solomon Islands
Source: Malar J. 2012 Mar 30;11:101. doi: 10.1186/1475-2875-11-101 (PMC3359162; doi:10.1186/1475-2875-11-101)
Supplement: Additional file 1 — List of staff whose contribution to the mass blood survey is acknowledged and appreciated. [file 1475-2875-11-101-S1.DOCX]

The authors would like to sincerely thank the following staff that assisted with the collection and processing of data during the mass blood survey.

AMI Staff:

J.Staley

B. McPherson

G. Birrell

T.Whyte

S.Tsai

C.Atkins

B.Cooper

A.Baron

J.Beckett

M.Edstein

J.Pickering

C.Mostyn

C.McCormack

D.MacKenzie

S.Smith

N.Waters

K.Gresty

K.Rowcliffe

S.Corcoran

W.Sharrock

R.Perrin

Solomon Islands NVBDCP Staff - Microscopy and Entomology:

P.Toriana

R.Seni

R.Gigini

H.Lily

H. Bugoro

J.Manesonia

A.Apairamo

L.Landry

E.Doedoke

B. Hou

A.H. Fafale

C. Iro’ofa
